# Existing psychological supportive care interventions for cervical cancer patients: a systematic review and meta-analysis

**DOI:** 10.1186/s12889-024-18634-3

**Published:** 2024-05-28

**Authors:** Kamala Dhakal, Changying Chen, Panpan Wang, Joanes Faustine Mboineki, Bibhav Adhikari

**Affiliations:** 1 School of Nursing and Health, Zhengzhou University, Jianshe Dong Lu, Zhengzhou, Henan 450000 China; 2 The First Affiliated Hospital of Zhengzhou University, Zhengzhou, Henan China; 3 Maharjgunj Nursing Campus, Maharajgunj, Kathmandu Nepal; 4 Henan Institute of Hospital Management, Zhengzhou, Henan China; 5 School of Nursing and Public Health, University of Dodoma, Dodoma, Tanzania; 6 Little Angels’ College of Management, Hattiban, Lalitpur, Nepal

**Keywords:** Cervical cancer, Psychology, Supportive care, Needs, Anxiety, Depression

## Abstract

Cervical cancer patients commonly experience psychological supportive care needs, necessitating diverse interventions to enhance psychological well-being and alleviate physical symptoms. This systematic review, covering English-published articles from January 1999 to April 2023, assessed the impact of psychological supportive care interventions on anxiety and depression. Twenty-Six studies, including 11,638 patients, were analyzed, comprising randomized controlled trials; quasi-experimental, and pre-post-test designs from PubMed; Science Direct; Wiley online library; Google Scholar; Cochrane Library; and JSTOR. The extraction of data was done by two independent authors and a third independent author checked the data extraction. The Preferred Reporting Items for Systematic Reviews and Meta-Analyses (PRISMA), 2020 statement was adopted. The population, intervention, comparator, and outcomes (PICO) search strategy was applied. Effective Public Health Practice Project (EPHPP) tool was used to assess the quality of selected articles. Various interventions, such as psychological nursing, exercise, counselling, psycho-curative approaches, peer and family education, psychotherapy, and medication, were identified. Two studies incorporated homework sessions, predominantly administered by nursing staff. Self-Rating Depression Scale (SDS) and Self-Rating Anxiety Scale (SAS) were commonly used instruments. Statistical analysis revealed a significant difference in anxiety and depression scores between treatment and control groups (*p* < 0.005) post-intervention across all studies. A subsequent meta-analysis of eight homogeneous studies, utilizing a random-effects model, showed a moderate-to-high overall effect size (1.35, 95% CI: 0.75 to 1.94), indicating a statistically significant positive impact. Various studies exhibited variability in effect sizes ranging from low to high. While the meta-analysis included 936 participants, the forest plot visually represents individual study effect sizes and the combined effect size. Preliminary evidence supports the positive impact of psychological supportive care interventions on cervical cancer outcomes, urging further research, especially exploring long-term effects and employing rigorous study designs.

## Background

Supportive care (SC) is the provision of the necessary services for those living with or affected by cancer to meet their informational, emotional, spiritual, social, psychological, and physical needs during their diagnostic, treatment, or follow-up phases encompassing issues of health promotion and prevention, survivorship, palliation, and bereavement [1]. Supportive care Needs (SCNs) are the necessities of individual patient care: Related to their symptoms, side-effects management, adjustment, dealing with disease, optimization of acceptance, informed decision-making, and minimization of practical deficits [2]. Psychological SCNs include the following problems: anxiety, feeling down or depressed, feelings of sadness, fears about cancer spreading, worry that the results of treatment are beyond your control, uncertainty about the future, learning to feel in control of your situation, keeping a positive outlook, feelings about death and dying Concerns about the worries of those close to you [3].

Psychological supportive care intervention is any set of activities that are used to change behaviors, emotions, or cognitions of a person who suffered from any kind of psychological problems [4]. It is also called psychological treatments, can be highly effective for many mental health conditions, particularly anxiety and depression [4]. It can be delivered by trained and supervised non-specialists in single or combined form. It is found in different form like cognitive behavioral therapy, psychotherapy, exercise, play therapy, counselling, group therapy [4, 5], medication [6] etc.

In comparison with other gynecological cancer patients, cervical cancer (CC) patients face manifold problems such as psychological and social distress, spiritual suffering, irritability, memory loss, worse emotional distress, and poor quality of life [7]. According to a hospital-based cross-sectional study in Ethiopia, 79.7% of CC patients experienced anxiety and 47% of CC patients felt depression [8]. CC patients in Seoul shared that they needed psychological supportive care to cope with moderate and severe depression (11.08 ± 5.06) [9]. CC patients under treatment in a cancer hospital in Zambia needed psychological supportive care: 80% of patients reported depressive symptoms, 78% moderate, 18% mild and 4% severe [10]. The most common complaint among CC patients was the loss of concentration (85.0%) in Mainland China [11]. With the help of supportive care intervention (SC), patients and family members can manage these disease-related problems comprehensively and holistically during the disease course [12, 13].

This systematic literature review aimed to synthesize currently available evidence about the psychological supportive care intervention for psychological SCNs (anxiety and depression) of women living with cervical cancer, driven by the following research questions:

- What is the current evidence about the different psychological supportive care interventions for psychological SCNs (anxiety and depression) in women living with CC?
- What is the effect of different psychological supportive care interventions on psychological SCNs (anxiety and depression) among women living with CC?

### Significance of the study

This study will help researchers to implement the most effective psychological intervention that will result in a precise outcome.

## Methods

### Study selection criteria and search strategy

This study is a systematic review and meta-analysis which conducted to examine the effect of the psychological supportive care intervention to resolve psychological supportive care needs; “anxiety and depression” among CC patients. Psychological supportive care needs mean the following ten items as mentioned in psychological supportive care need domain of Supportive Care Need Survey –Short Form 34 (SCNS-SF 34). These items are as follows: anxiety, depression, feeling sad, fear of spreading the cancer, tension that health condition will be beyond control after the treatment, uncertainty about future, feeling of being yourself under control of the situation, maintaining positive thinking, feeling tension of death and dying, worry about your loved one [14]. Authors tried to explore psychological supportive care intervention in any form (exercise, counselling, psychotherapy, empowerment education, medication) for the management of anxiety and depression among those mentioned ten psychological supportive care needs items.

This review was registered in PROSPERO International’s prospective register of systematic reviews with ID No CRD42023164594 (https://www.crd.york.ac.uk/prospero/#myprospero).

Preferred Reporting Items for Systematic Reviews and Meta-Analyses (PRISMA) updated guidelines [15] and the recommendations of the Cochrane Collaboration [16] were used to conduct this systematic review. The methods applied for this systematic review are similar to the guidelines detailed in the PRISMA, 2020 checklist [15]. Six electronic databases (PubMed, Science Direct, Willey online library, Cochrane, Google Scholar, and JSTOR) were searched through a two-step systematic search strategy that was devised to identify studies employing qualitative and/or quantitative methods. A wide range of keywords and free text terms were used to increase the inclusiveness and sensitivity of the searches. Mandalay was used as the automation tool in this review. Pre-specified selection criteria were applied to all records identified. Reference lists of all full-text articles were be also examined for any studies that might have been overlooked. Electronic searches began on 20th March 2023 and were completed on 30th April 2023. We searched the specified databases since 1999 Jan to 2023 April. English-published articles randomized control trials (RCTs), quasi-experimental studies, and one group pre-test-posttest were included in the study. Search terms were psychological, supportive care, intervention, anxiety, depression, and cervical cancer. The population, intervention, comparator, and outcomes (PICO) search strategy was applied (Refer to Table 1).

**Table 1:** Application of the PICO search strategy

PICO strategy	Description
Population	A woman living with cervical cancer within the age group of 18 years and above
Intervention	Psychological intervention
Comparator	Patients receiving placebo or usual care or wait listed
Outcome	Primary outcome: Anxiety, Depression Secondary outcome: Stress, quality of life, cortisol level, self-efficacy, sexual function, sleep status, and fatigue
Study design	RCT, Quasi experimental, One group pre-post test

### Example of search strategy from PubMed Central (PMC)

The search words were: psychological AND supportive care AND intervention AND anxiety AND depression AND cervix AND cervical AND cancer. The following search strategies were used: ((“psychologic“[All Fields] OR “psychological“[All Fields] OR “psychologically“[All Fields] OR “psychologization“[All Fields] OR “psychologized“[All Fields] OR “psychologizing“[All Fields]) AND (“anxiety“[MeSH Terms] OR “anxiety“[All Fields] OR “anxieties“[All Fields] OR “anxiety s“[All Fields]) AND (“depressed“[All Fields] OR “depression“[MeSH Terms] OR “depression“[All Fields] OR “depressions“[All Fields] OR “depression s“[All Fields] OR “depressive disorder“[MeSH Terms] OR (“depressive“[All Fields] AND “disorder“[All Fields]) OR “depressive disorder“[All Fields] OR “depressivity“[All Fields] OR “depressive“[All Fields] OR “depressively“[All Fields] OR “depressiveness“[All Fields] OR “depressives“[All Fields]) AND ((“support“[All Fields] OR “support s“[All Fields] OR “supported“[All Fields] OR “supporter“[All Fields] OR “supporter s“[All Fields] OR “supporters“[All Fields] OR “supporting“[All Fields] OR “supportive“[All Fields] OR “supportiveness“[All Fields] OR “supports“[All Fields]) AND “care“[All Fields]) AND (“intervention s“[All Fields] OR “interventions“[All Fields] OR “interventive“[All Fields] OR “methods“[MeSH Terms] OR “methods“[All Fields] OR “intervention“[All Fields] OR “interventional“[All Fields]) AND (“uterine cervical neoplasms“[MeSH Terms] OR (“uterine“[All Fields] AND “cervical“[All Fields] AND “neoplasms“[All Fields]) OR “uterine cervical neoplasms“[All Fields] OR (“cervical“[All Fields] AND “cancer“[All Fields]) OR “cervical cancer“[All Fields])). The search was only limited to publication dates from 1st January 1999 to 2023 April. The search was performed on 23rd April 2023.

### Inclusion criteria

Randomized control trial, experimental design, and one group pre-post studies conducted among cervical cancer targeting psychological problems and full-text articles in the English language were included in the study (Refer to Table 1).

#### Exclusion criteria

Review studies, qualitative studies, cross-sectional quantitative studies, commentaries, letters, pilot studies, preprint articles, clinical trials with international trial registries but unpublished and study protocols were excluded from the study. If the content of the selected article did not match the inclusion criteria after a thorough reading, those articles were not included in the study. Studies were excluded if full papers could not be found.

#### Intervention

The intervention involved the training of CC patient healthcare professionals/trainers/psychologists through health education, and physical and psychological exercise targeting to address the psychological problems.

### Comparator(s)/Control

The patients with usual (regular) care was considered as control group on the studies review.

### Outcome measures

The outcome measures for studies included in this review were the reduction of anxiety and depression after getting involved in the targeted intervention as a primary outcome.

### Study selection and data extraction procedures

Two authors (KD and JFM) independently screened the titles and abstracts of the articles for their and discrepancies around inclusion were resolved by discussion with the third author (BA) following a two-stage process. The initial screening stage resulted in a shortlist of articles including titles and abstracts. In the second stage, the screening process involved retrieval of articles in full- text, whereby the two co-authors independently assessed all articles for eligibility against selection criteria until a consensus was reached. Data extraction tables were specifically developed for this review, pilot-tested on three randomly selected studies of the final sample, and refined accordingly. After eliminating the duplicates, two authors (KD, BA) independently extracted data from each of the eligible reviews into a purpose-built, standard data extraction form and a third independent person (CC) checked the data extraction [17].

### Assessment of risk of bias in included studies

It was evaluated using an Effective Public Health Practice Project (EPHPP) tool by two review authors (KD and JFM) independently. It contains 8 components: selection bias, study design, confounders, blinding, data collection method, withdrawals/ dropouts, intervention integrity and analyses. Each component is rated as weak (1 point), moderate (2 points), and strong component (3 points). The maximum total score per study is 3.00. Based on their total score, the quality of studies is rated as weak (1.00- 1.50), moderate (1.51–2.50), or strong (2.51-3.00)0.31 The records underwent final assessment according to the EPHPP tool along with established inclusion and exclusion criteria [18, 19].

Any disagreement was resolved by involving a third reviewer (BA). We contacted the authors to obtain any missing data. The extracted data included the following information:

• Publication details: author, year, country • Study characteristics: Total number of participants, mean age of participants, type, and stage of cancer.
• Intervention design: Content of the intervention, duration of intervention, the total number of sessions, duration of each session,
• Outcomes: Type of outcome to be measured, timing, frequency, and duration of follow-up for each outcome, outcome measurement tool.

### Assessment of risk of bias across studies

We tried to reduce the risk of publication bias by searching international trial registries and unpublished studies. Where we doubted reporting bias, we started to contact study authors to request them to make availability of missing out- come data.

### Data analysis

Due to data heterogeneity, a systematic review was conducted to explore the relevant intervention and its characteristics like design, sample size, outcome measure, and outcomes.

After initial screening, the full texts relevant to the topic were reviewed independently by 2 authors (JFM and KD). Extraction of data was performed and entered in a data charting form in Microsoft Excel. Any emerged disagreements concerning inclusion and exclusion from the final review, and the third author (BA) got involved. After the data were entered into a data charting form, the authors (JFM and KD) reviewed the data to identify the review’s key focus areas. The results of the review are reported according to the PRISMA Statement [15].

The studies included in the revision were first examined for descriptions of the interventions and qualitative synthesis. A narrative synthesis was done after listing down the components of the intervention. The findings of different studies were described and combined into the text of the review by examining the similarities and differences among the results of all reviewed studies. The population characteristics, design of the study, intervention, instrument used and outcome of reviewed studies were identified. As well the patterns and relationship in the data within and between the reviewed studies were also investigated [20, 21].

Quality of each reviewed study was assessed by following the guidelines of quality assessment tool for quantitative studies developed by EPHPP. According to EPHPP guidelines quality of each study is calculated on following eight items: selection bias, study design, confounders, blinding, data collection methods, withdrawals/dropouts, intervention integrity and analysis by using three points Likert scale. As per this liker scale score 1 indicates strong study, score 2 indicates moderate study and score 3 indicates weak study in above mentioned eight parameters [18, 19, 22].

## Results

### Study characteristics

**Fig. 1:**
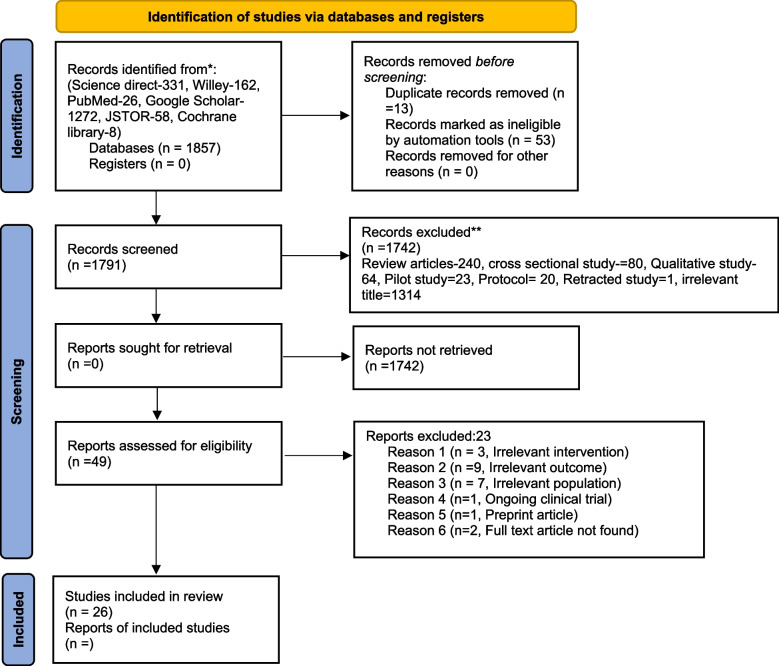
Study flow diagram based on PRISMA, 2020

On the first electronic literature search a total of 1857 records were identified through six different databases and it was reduced to 1791 after duplicates were removed and records were marked as ineligible by automation tools. After screening titles and abstracts, the records were further reduced to 49 for full-text eligibility while 1742 records were excluded because their titles and abstracts did not conform to the topic or the study designs. After thorough and detailed readings of illegible full-text articles, 26 articles were included in this review. Out of these, 23 articles were excluded because 3 articles had an irrelevant intervention, 9 articles had irrelevant outcomes, 7 articles had an irrelevant population, 1 article had an ongoing clinical trial, 1 article had a preprint article and 2 articles full text had not found (Refer to Fig. 1).

### Total participants, mean age, design, country, and setting

Among twenty-six interventional studies included in this review, thirteen studies were RCTs [6, 23–34], twelve studies were quasi-experimental design [35–46] and one study was one group pre-posttest design [47] with 11,638 cervical cancer patients from 6 different countries (China, Indonesia, Turkey, Zambia, USA and India). Out of twenty-six reviewed articles, majority; eighteen studies were from china, two form Indonesia, one from Turkey, one from Zambia, one USA and one from India. All studies were conducted in hospitals. The mean age of the respondents was between 34.15 ± 10.18 to 66.7 ± 4.5 in the intervention group and between 36.57 ± 11.42 to 65.7 ± 4.1 in the control group whereas the mean age of the respondents was not mentioned in seven studies (Refer to Table 2).

**Table 2:** Characteristics of included studies

SN	Country, Year & Author	Design	Study size T and C		Mean age in years (T/C)	Stage of cancer & IT	Intervention & given person		Number period and place of intervention	Scales, Outcomes and Quality rating EPHPP
			T	C			Treatment	Control		
1.	Indonesia, 2021 [29]	QE	15	15	NM	I-III DCT	Progressive muscle relaxation and physical exercise the Provider- experts in the field of sports who have certificates in the field of sports.	NM	1st week - Progressive muscle relaxation therapy 2nd week -Physical exercise 3rd week. -Physical exercise and progressive muscle relaxation therapy Total = 21 sessions 30 min of each session NM	HADs & PFS - There was a significant difference between depression level with t = 3.552 (*p* < 0.05) and anxiety level with t = 11.297 (*p* < 0.05) after physical exercise. Strong
2.	China, 2021 [30]	QE	55	54	53.29 ± 12.49 54.37 ± 13.08	I-IIa DCT The inter- venation was performed from 1 day before surgery to 10 days after surgery	Comprehensive psychological intervention combined with conventional nursing (Comprehensive cognitive intervention, Behavioral intervention (PMRT), Emotional intervention, Social support intervention Providers - Nursing staff	Conventional nursing intervention ( preoperative guidance through making rounds, active cooperation during surgery, postoperative health education and discharge guidance	1–10 days (total 10 sessions, but duration is not mentioned for control group) 1–10 days (total 10 sessions) 1-hour duration each day (10 min for PMRT within this 1 h) Hospital ward	HADs, SSRS, HHS, PSQI and Levels of Cortisol (Cor) and interleukin-2 (IL-2) -The comprehensive group had lower scores of HADS-A and (HADS-D), higher scores of SSRS, intimate relationship than the conventional group (*P* < 0.05). Strong
3.	China, 2021 [16]	RCT	43	43	(59.66 ± 3.42) 58.24 ± 4.17)	Pathology subtypes (Adenocarcinoma Squamous cell carcinoma Adeno-squamous carcinoma) DCT	Bundled nursing care (health education about cervical cancer, psychological intervention, Nursing care of chemotherapy complications, Home care guidance after chemotherapy, and Periodic return visits) and supported peer education Provider - 1 chief physician, 1 chief nurse and 3 nurses	Bundled nursing care (health education about cervical cancer, psychological intervention, Nursing care of chemotherapy complications, Home care guidance after chemotherapy, and Periodic return visits)	NM NM Hospital ward	SDS, SAS and GSES. -The scores of depression and anxiety in two groups were remarkably decreased after intervention compared with prior-treatment, and the range of decrease in the observation group was critically greater than that in control group (*P* < 0.05). Strong
4	China, 2021 [42]	RCT	52	52	66.7 ± 4.5 65.7 ± 4.1	FIGO (Ia–Iib) Pathological type (Squamous cell carcinoma, Adeno-carcinoma, Adenos-quamous carcinoma Before Hysterectomy without receiving any cancer treatment	Routine nursing care & Whole-course standardized nursing and humanistic care (Preoperative nursing, Intraoperative nursing, Postoperative nursing and Discharge guidance Provider: Nursing staff	Routine nursing care	NM NM Hospital ward	Chalder Fatigue Scale (CFQ), PSQI, BDI, BAI, EORTC-C30 and HHI - The patients’ negative emotions and level of hope were significantly improved in intervention group. Strong
5.	Indonesia, 2018 [21]	RCT	16	16	Not mentioned	IB - IVB1 DCT	Supportive psychotherapy (it is group therapy) Details not mentioned Provider- Psychotherapist	Common psychotherapy	NM NM NM	Cortisol value, Distress thermometer score, and HAM-D17 After the intervention of psychotherapy in the treatment group decreased HAM-D17 score, the average decline 7.53 (SB 3.34). The mean decreasing in the control group was 3.98 (SB 2.85). There is a significant difference in mean reduction in HAM-D17 scores on treatment and control groups with *p* = 0.003 (*p* < 0.005). Strong
6.	USA, 2015 [22]	RCT	115	89	44.9 ± 9.5 44.6 ± 9.7	I-IVA Had com- plated definitive treatment at least 2 months earlier Home based	Psychosocial telephone counseling Session I, a QOL/psychosocial interview Sessions II to IV managing stress and emotions, health and well- ness, and managing relationship and sexuality concerns Homework after each session Provider- Counsellor	Usual care	PTC a precall (5 min) to reintroduce the purpose of the intervention schedule session I, a QOL/psychosocial interview (generally 60 min). Sessions II to IV (range, 20 to 60 min) included topics of managing stress and emotions, health and well- ness, and managing relationship and sexuality concerns Homework after each session 1-month duration Usual care not mentioned Home	The Patient-Reported Outcomes Measurement Information System (PROMIS) Emotional distress depression short form The Brief Symptom Inventory (BSI-18) The Gynecologic Problems Checklist (GPC) & FACT-Cx Respondents receiving PTC had significantly improved depression and improved gynecologic and cancer-specific concerns at 4 months compared with UC participants (all *P* < 0.05); significant differences in gynecologic and cancer-specific concerns (*P* < 0 0.05) were continued at 9 months. Strong
7.	India, 2021 [43]	RCT Unblended, randomized two-arm study	24	24	NM	I = IV DCT	Yoga nidra Relaxation, resolve, rotation of consciousness, breath awareness, image visualization; and resolve Volunteers of the yoga nidra after getting training from trained yogi	NM	4 weeks (28 sessions) 23 min’ duration of each session Hospital ward	The stress questionnaire comprising the psychological, physical, social, and financial problems and grading and categorization stress indices Compared to the control group, the stress was significantly less in the groups that practiced yoga nidra (79.46 vs. 64.42) (*P* < 0.0001) Strong
8.	Zambia, 2021 [46]	RCT Double blinded	22	22	NM	NM After dx. of CC before the start of CC Rx	Supportive group therapy (Detail of supportive care is not mentioned) Provider- Psychologist	Usual support and care of staff and their families	4 weeks (28 sessions) One-hour session NM	Hopelessness in Illness Questionnaire (HAI) The intervention group had a greater reduction in HAI scores from (*p* = 0.621 to *p* = 0.368) in comparison to the control group (*p* = 0.707 to *p* = 0.683). Strong
9.	China, 2020 [25]	RCT	48	48	48.4 ± 7.4 48.6 ± 6.8	IA2- IB2 During CC surgery	Comprehensive nursing care (Rehabilitation training, Psychological nursing, Information about their disease, Diet intervention) Nursing staff	Routine nursing care ( information and guidance about relevant drugs, recommended to eat a balanced diet, educated about cervical cancer and health and informed about hospital telephone number)	Rehabilitation training − 3 times a day, Others not mentioned Hospital ward	Morisky Medication Adherence Scale (MMAS-8), MOS 36-Item & Short-Form Health Survey (SF- 36) The respondents’ in observation group got significantly higher MMAS-8 and SF-36 scores than the control group and showed significantly higher total nursing satisfaction than the control group (all *P* < 0.05). Strong
10.	China, 2021 [26]	RCT	66	66	44.4 ± 8.2 45.5 ± 7.9	IA-IIB During CC surgery	Regular continuous nursing (regular discharge guidance) & Collaborative continuous nursing (giving guidance on diet, medication, pain control, psychological health, self-care, review time and recognition of postoperative complications) Provider- team of 10 medical staff (3 doctors (1 chief physician, 2 attending physicians), 1 dietitian, 1 pharmacist, 1 psychological counselor, 1 sex counselor, 1 head nurse and 2 nurses in charge)	Regular continuous nursing ( regular discharge guidance)	3 months Total number and frequency of intervention are not mentioned. Hospital ward	SAS, SDS, MOS SF-36, PFS & PSQI The anxiety and depression scores of patients in the observation group were significantly lower than those in the control group (both *P* < 0.001). Strong
11.	China, 2020 [27]	RCT	35	34	34.15 ± 10.18 36.57 ± 11.42	NM During CC surgery to 6 months after surgery	Empowerment education-based nursing interventions (Clarify problems, Expression, Set goals, Planning) Provider- Nursing staff	Conventional nursing interventions ( health education, psychological counseling, diet intervention, guidance on postoperative recovery, and sexual life)	6 months Total number and frequency of intervention are not mentioned. Hospital ward	Female Sexual Function Index (FSFI), SDS, A home-made questionnaire which covers what patients do or do not know about cervical cancer, Self-care ability determination scale (ES- CA) & EORTCQ- LQ-C30 The SDS scores in the OG were lower than they were in the CG (*P* < 0.05). Strong
12.	Indonesia, 2019 [28]	RCT Double blinded	15	15	NM	IIB-IV DCT	Standard therapy/ chemo radiation, & Psych curative (Cognitive, spiritual, social, and physical support). It is done in group Provider of psych curative is NM Detail of psych curative is NM	Standard therapy/ chemo radiation,	1-month duration Four meetings (once a week), 16 total sessions, Each one 60 min Discussion and exploration of the experience that patient had during the previous psych curative in each session The subjects performed an individual psych curative three times a week at home with a psych curative guide book given to them. Researchers visit each subject’s house once a week in order to monitor and motivate the subject. Hospital ward and home	Serum cortisol level, Depression, anxiety and stress scale 42 (DASS 42) and WHO quality of life questionnaire The difference mean test result of anxiety level, from the controlled group and uncontrolled group showed a significant difference (*p* < 0.05) Strong
13.	China, 2022 [18]	RCT	55	55	NM	Ia- Ib During radical CC surgery	Fast-track surgical nursing (Psychological counseling, preoperative preparation, intraoperative nursing, and postoperative recovery instruction) Provider- Group of nursing staff	Routine perioperative nursing ( health education, medication guidance, hospitalization environment, and daily diet intervention)	NM Hospital ward	Karnofsky per- formance status (KPS), SDS, SAS, Quality of life (QOL) for patients with cancer, Immunoglobulin A (IgA), Immunoglobulin M (IgM), and Immunoglobulin G (IgG). Patients in the research group after intervention had significantly lower SAS and SDS scores compared to the reference group (*P* < 0.001). Strong
14.	China, 2020 [19]	RCT Double blinded	312(Three group, each group-104)	105	48.53 ± 10.0 48.11 ± 10.38 47.07 ± 10.08 46.27 ± 10.83	I- II During laparoscopic modified radical hysterectomy of CC	Racemic ketamine group received 50 ml 0.5 mg/kg racemic ketamine by intravenous injection after 1 h of analgesia High- dose S-ketamine group received 50 ml 0.5 mg/kg S-ketamine by intravenous injection after 1 h of analgesia Low-dose S-ketamine group received 50 ml 0.25 mg/kg S-ketamine by intravenous injection after 1 h of analgesia. Provider- Anesthesiologists & Surgeons	Patients received 50 ml normal saline by intravenous injection after 1 h of analgesia;	7 days Total number and frequency of intervention are not mentioned Hospital ward	HAMD-17, Visual Analogue Score (VAS) for pain & Serum levels of BDNF and 5-HT The high-dose S-ketamine group showed significantly lower VAS and HAMD-17 scores than all other groups at 1 day and 3 days postoperatively. Strong
15.	China, 2021 [45]	RCT	116	116	NM	IIIa- IVa Postoperative CC patients	Basic care combined with Chinese herbal medicine treatment Psychological care treatment (proper communication, music therapy, conversation method, emotional catharsis) Provider- Nursing staffs	Basic care combined with Chinese herbal medicine treatment	Chinese herbal (One dose a day, Divided into morning and evening. Fifteen days was a course of treatment, followed by four courses of treatment. (60 session/ 60 days) NM about psychological care Hospital ward	Tumor volume was calculated as length- ∗width ∗radius, WHO solid tumor efficacy criteria Visual analogue scale (VAS) Symptom Checklist 90 (SCL-90), Health related Quality of life scores SF-36 scale, Serum CRP and IFN-c The total effective rate of the study group was higher than that of the control group. VAS scores in the study group were significantly lower than those in the control group 30 and 60 days after treatment. The SCL-90 scores of the study group after treatment were lower than those of the control group. Strong
16.	China, 2020 [48]	QE	55	54	48.52 ± 1.28 48.49 ± 1.22	I-II During concurrent radio chemotherapy	Conventional nursing care & Mindfulness-based stress reduction (mindful breathing, mindful meditation, body scan, walking meditation, eight-sectioned exercise, emotional regulation and homework after each session) Provider- NM	Conventional nursing care ( patient education for the disease, dietary guidance, prevention and treatment of adverse reactions, and positive social as well as psycho- logical support)	Total 6 weeks: one session per day, total 42 session Week 1- “mindful breathing” for 50 min, homework for MFB every day Week 2: “mindful meditation”0.50 min, homework for MFB and meditation exercises every day Week 3: “body scan” for 50 min, following the classical music Autumn Moon Over Han Palace, they scanned their body top-to-bottom and paid attention to any feelings. If some dis- comfort occurs, they were asked to imagine that it disappears with the breath. Homework of mindful-ness breathing and meditation and body scan exercises every day Week 4: “walking meditation”. 50 min class, following the classical music Dialog Between Fisherman and Woodcutter being played, they took a mindful breath and then imagine that they are walking for the first time and feeling the movement of the body and contact with the ground. The homework was using mindfulness breathing, meditation and body scanning and walking meditation exercises every day. Week 5: “eight-sectioned exercise”. For 50 min, they were assisted to learn the first three sections and pay attention to any changes in emotions, physical sensations and breathing. The homework was given about mindfulness breathing, meditation, body scanning, walking meditation and the last sections every day Week 6: The theme of the intervention was “emotional regulation” for 20 min Hospital ward	SAS, SDS, Anderson Symptom Assessment Scale (MDASI), Self-perceived burden scale (SPBS), PFS, & FACT-CX After nursing care, patients in the OG reported lower SAS and SDS scores than those in the CG (*P* < 0.05) Strong
17.	China, 2021 [34]	QE	80	61	41.22 ± 2.33 40.98 ± 2.82	II-IV During postoperative chemotherapy period	Routine nursing intervention & Psychological nursing (The establishment of a psychological intervention team, Psychological evaluation, Psychological intervention: Hospital intervention; Nursing intervention outside the hospital, Periodic evaluations) Provider- Team of surgeons, psychologists and primary nurses	Routine nursing intervention ( health education, functional training, the prevention of complications, and regular follow-up in addition to the treatment)	90 days’ duration Total sessions and frequency of sessions is not mentioned Hospital ward and home	EROTC-QLQ-C30 & SAS The patients’ SAS scores in the two groups before the intervention were not significantly different (*P* > 0.05). Strong
18.	Turkey, 2013 [35]	QE	20	20	49.97 ± 11.31 This is as a whole, not written separately for T and C group	NM During third and fourth chemotherapy cycle	Usual care & Back massage (8steps) Provider researcher	Usual care	One day – one session (60–75 min for each session (back massage for 15 min before the infusion, between 25th and 40th minutes of each 1-hour period during the treatment and for 15 min at the end of the treatment in accordance with the duration of chemotherapy. Chemotherapy cycles administered to the patients included in the study were minimum 2-hour and maximum 3-hour cycles. Thus, patients had a 60-minute back massage during the 2-hour cycle and a 75-minute back massage during the 3-hour cycle) Single session Hospital ward	Spielberger State-Trait Anxiety Inventory (STAI), Brief Fatigue Inventory (BFI) The mean anxiety scores of the patients in the intervention group decreased right after the massage provided during chemotherapy (*p* = 0.109; effect size = 0.37) Strong
19.	China, 2021 [36]	QE	50	50	53.23 ± 3.23 NM separately for T & C	IIA- IV During percutaneous arterial chemotherapy Rx for CC	Routine nursing and comprehensive nursing (psychological nursing, health education, diet management, post-operative care, social support was Provider- Nursing staffs	Routine nursing ( observing their condition, providing symptomatic treatment, strictly following the principle of aseptic operation, avoiding infection)	NM Hospital ward	SDS & SAS After nursing intervention, the incidence of complications and the scores of depression and anxiety of the experimental group were significantly lower compared with the control group, *P* < 0.05. Strong
20.	China, 2021 [41]	One group pre post test	96	No control group	40.5 ± 7.15	I-IV During postoperative treatment of CC	Traditional Chinese medicine (TCM) Psychological Intervention Provider- Nurses	No control group	TCM 30 sessions (Total 3 courses 1 courses contain 10 continuous day administration of TCM, in each month, ) 3 months’ course Psychological intervention (NM the total session, duration and frequency) Hospital ward	SDS, SAS, Changes of T-Lymphocyte Subset-Related Indicators (CD3+, CD4+, CD8+, and CD4+/CD8+) and blood routine- related indicators, Coagulation Function & Tumor Marker (CA125) Examination. After treatment, depression and anxiety were significantly reduced and the patient’s quality of life significantly improved. Weak
21.	China, 2022 [37]	QE	60	60	50.24 ± 6.49 49.42 ± 6.49	Squamous carcinoma Adenocarcinoma Adenosquamous carcinoma During comprehensive anti-cancer treatment	Whole-course high-quality care (Before, During and after comprehensive treatment) combined with network continuation care interventions (Using WeChat). Provider- Team of medical and nursing staffs	Conventional care interventions ( verbal health education about cervical cancer; they were informed of the precautions and examination items during surgery and radiotherapy; they were advised to complete daily basic care; they were strictly supervised to take medication and receive treatment on time; the hygiene and room temperature of wards and treatment rooms were maintained; and the changes of patients’ vital signs were strictly monitored and recorded)	Total session, frequency and duration not mentioned. Hospital ward	Comprehensive treatment cognitive score, Comprehensive treatment compliance score, Incidence of adverse reactions, Quality of life questionnaire (QLQ-C30) score, SAS, SDS & Nursing satisfaction After care, SAS and SDS scores were lower in both groups than before care, and were lower in the joint group than in the regular group (*P* < 0.05). Strong
22.	China, 2022 [38]	QE	131	105	38.85 ± 7.03 39.15 ± 6.68	Squamous carcinoma Adenocarcinoma Adenosquamous carcinoma During radical laparoscopic CC Rx	Psychology Group Nursing Method (convention group, the psychology group adopted the family- oriented enabling psychological nursing. Family-oriented enabling nursing was given as follows: through small lectures and according to the educational level of the family members, the preoperative nursing staff conducted the education of professional knowledge related to cervical cancer and surgical treatment for the family members and patients Intervention provider = Nursing staff	Convention Group Nursing Method ( preoperative health education, preoperative preparation, preoperative visit, intraoperative nursing cooperation, postoperative basic nursing, keeping the ward clean and ventilated, and disinfecting regularly	Once a week 1 h for psychology group Other not mentioned Hospital ward	PTSD Checklist-Civilian Version (PCL-C), Fear of Progression Questionnaire-Short Form (FoP- QSF) & The Cancer Rehabilitation Evaluation System-Short Form (CARES-SF) The PCL-C score, FoP-Q-SF score, and CARES-SF score decreased in the psychology group and the convention group (*P* < 0.05) and the decreases of those three scores were more obvious in the psychology group Strong
23.	China, 2019 [39]	QE	77	77	49.73 ± 9.12 51.27 ± 8.78	Ib2- IIb During Neoadjuvant chemotherapy(NACT)	Psychological nursing on the basis of routine nursing (Pleasurable activities, Psycho- logical resolution and persuasion, Sharing of experiences Provider- NM	Routine nursing care ( Clinical disease monitoring, Improvements of environ- mental conditions, Dietary guidance, Medication guidance)	Total duration 2 weeks Total sessions = 14 (Pleasurable activities - once a week for about 15 min each time, Sharing of experiences once a week for about 30 min each time Hospital ward	NACT efficacy evaluation, Quality of life SF-36, SDS, SAS, Mean health status, scoring SCL-90 scale After the nursing intervention, the scores of life quality, anxiety and depression, mental health of the observation group were significantly improved and were better than those of the control group (all *P* < 0.05), especially in terms of the scores of emotional function (*P* < 0.001) and somatic pain (*P* = 0.012). Strong
24.	China, 2021 [40]	QE	78	73	42.37 ± 10.39 41.98 ± 11.01	Ia- IIa During laparoscopic radical hysterectomy of CC	Conventional nursing & Crisis intervention nursing: [1] Establishment of a crisis intervention team [2] Admission assessment [3] Crisis intervention countermeasures ① Cognition ②Psychology ③ Behaviors ④ Social support [4] Intervention measures Provider- Team of surgeons, nurses, and psychological counselors	Conventional nursing intervention: (health training, basic nursing care, psychological nursing care, functional exercise and prevention of complications).	6 months’ study Total session, frequency and duration of each session not mentioned. Hospital ward	HAMA, HAMD, The cancer self- efficacy scale (SUPPH), Psychological crisis level assessment scale & The Herth Hope Index The scores of HAMA, HAMD, self-efficacy, psychological crisis and hope degree in the two groups were remarkably improved after intervention compared with before intervention (all *P* < 0.05), and the improvement of each index in observation group was obviously superior to that in control group (*P* < 0.05). Strong
25.	China, 2022 [31]	QE	43	37	44:05 ± 6:91 41:35 ± 7:09	NM During concurrent chemo radiotherapy treatment For CC	Case management model: 1.Preparation before nursing, establish a case management team, and the team members are the backbone of the department. 2.The implementation of the case management nursing model. 3.Personalized traditional Chinese medicine characteristic nursing. Provider- Team of nursing and medical staff	General psychological support	Effect of intervention was evaluated 6 months ‘after intervention 6 months’ session Total session, frequency and duration of intervention is not mentioned. Hospital ward	HADs HADs score showed that anxiety and depression scores decreased after intervention in the experimental group, and the difference between two groups had significant after intervention (*P* < 0:05). Strong
26.	China, 2020 [32]	QE	77	77	49.73 ± 9.12 51.27 ± 8.78	Ib2- IIb During neoajuvant chemotherapy (NACT) for CC	Routine NACT nursing & Psychological nursing: [1] Emotional support [2] Psychological resolution and counseling = in-depth communication and conversation for 20–40 min, once a week [3] Experience exchange- once a week, about 30 min each time Provider- Nursing staff	Routine NACT nursing: 1. Close attention to the changes of the patients’ condition 2.Improvement of the environment 3.Dietary guidance 4.Guidance on medication	4 weeks’ duration Total 8 sessions 1. Psychological resolution and counseling = in-depth communication and conversation for 20–40 min, once a week 2. Experience exchange- once a week, about 30 min each time Hospital ward	MOS item short form health survey (SF-36), SDS and SAS After nursing intervention, anxiety and depression of the two groups were significantly improved as compared with those before nursing intervention (*P* < 0.05). And the improvement rating in the observation group was more better than the control group (*P* < 0.05), particularly in the fields of role-emotional (*P* < 0.001) Strong

*QE *Quasi experimental, *T *Treatment group, *C *Control group, *IT *Intervention timing, *PE *Physical exercise

*DCT *During cancer treatment, *NM *Not mentioned, *DX *Diagnosis, *RX *Treatment

### Study size

The sample size ranges from a minimum of 30 [24, 35] to a maximum of 417 [6]. An equal sample size was used in the treatment and control group in 16 studies and an unequal sample size was used in 10 studies.

### Stage of cancer and treatment trajectory

Regarding the stages of cancer, two studies included stage Ia–IIb cervical cancer [25, 48], two studies included stage I-II cervical cancer [6, 39], two studies included stage I-IV cervical cancer [30, 47], two studies included stage Ib2-IIb cervical cancer [38, 45], four studies included pathological subtype (squamous carcinoma, adenocarcinoma, adenosquamous carcinoma) of cervical cancer [23, 37, 43, 44] and three studies’ stages of cancer not mentioned [31, 34, 41] and another eleven studies were ranges from I- IV B1 remaining studies.

Regarding treatment trajectory, nine studies included patients receiving surgical treatment [6, 25, 27, 32–34, 36, 44, 46, 48], seven studies included patients receiving chemotherapy [23, 24, 28, 30, 35, 41, 42], three studies included patients receiving surgery plus chemotherapy [26, 40, 47], two studies included patients receiving concurrent radio chemotherapy [37, 39], two studies included patients receiving neoadjuvant chemotherapy [38, 45], one study included patients receiving comprehensive anti-cancer treatment [43], one study included patients completed the definitive treatment of cervical cancer before two months [29] and one study included patients they had just get a diagnosis of cervical cancer and before starting of cancer treatment [31].

### Interventions used in treatment and control group

The studies included in this review used a wide range of interventions for the treatment group. Among these, eleven studies used nursing intervention: comprehensive nursing [32, 36, 42], psychological nursing [38, 40, 44, 45], whole course standardized nursing [27], collaborative continuous nursing [33], empowerment education nursing [34], crisis intervention nursing [46] and perioperative nursing care [25]. Physical exercise was used in five studies: progressive muscle relaxation therapy [35, 36], yoga nidra [30], back massage [41] and mindfulness-based stress reduction [39]. Supportive group psychotherapy was used in two studies [28, 31]. Chinese herbal medicine and psychological care were used in two studies [26, 47]. Other interventions used in this review were: whole-course high-quality care [43], case management [37], drug therapy [6], psycho-curative [24], telephone counselling [29] and peer education [23]. One paper used drugs as psychological intervention for the management of anxiety and depression [6]. None of the articles explained about the co-intervention (received psychotherapy drugs).

Regarding the intervention for the control group, twenty-three studies mentioned the intervention used for this group but three studies [30, 35, 47] did not mention the intervention used for the control group. Homework session was mentioned by only two studies [29, 39] and the remaining other twenty-four studies did not mention that.

This review found heterogeneity in the design of the study, type of psychological intervention used; mode of delivery of intervention; intervention period; the number of sessions; follow-up period, intervention provider; control group; stage of cervical cancer; and instrument of outcome. measurement.

### The total session, duration of intervention, and provider of intervention

Among all studies, twelve studies explained in detail about total sessions, duration of each session, and total duration of each intervention [24, 26, 29–31, 35, 36, 38, 39, 41, 45, 47]. The range of sessions was minimum sessions were one [41] to maximum sessions sixty [26] and the mean sessions were twenty-four. The minimum duration of each session was twenty-three minutes [30], the maximum duration of each session was seventy-five minutes [41] and the mean duration of each session was 48.8 min. The total duration of each intervention is mentioned by eighteen studies [6, 24, 26, 29–31, 33–41, 45–47]. The minimum total duration for each intervention was one day [41], the maximum total duration for each intervention was 180 days [34, 37, 46] and the mean duration of each total intervention was 57.5 days. Whereas the remaining eight studies did not mention about total sessions, duration of each session, and total duration of each intervention [23, 25, 27, 28, 32, 42–44].

The providers of intervention were nursing staff in ten studies [25–27, 32, 34, 36, 38, 42, 44, 47] a team of doctors; nurses in four studies [23, 33, 37, 43] a team of surgeons; psychologists; nurses in two studies [40, 46] experts in the field of sports [35], psychotherapist [28], counsellor [29], volunteer [30], psychologist [31], a team of anthologist; surgeon [6] and researcher [41]. The remaining other three studies did not mention the provider of intervention [24, 39, 45].

### The scale used for the measurement of interventions

All studies used standardized and validated tools. The hospital Anxiety and Depression Scale (HADs) was used in three studies [35–37], Self-Rating Depression Scale (SDS) was used by ten studies [23, 25, 33, 38–40, 42, 43, 45, 47] Self-Rating Anxiety Scale (SAS) was used in ten studies [23, 25, 33, 34, 38, 39, 42, 43, 45, 47],, Hamilton psychiatric rating scale for depression (HAM-D17) was used in three studies [6, 28, 46].

### Psychological outcomes of interventions

There was a significant difference between anxiety and depression levels after psychological intervention (*p* < 0.05) in fifteen studies [23, 25, 27, 29, 33, 35–39, 42, 43, 45–47]. There was a significant difference between anxiety levels after psychological intervention (*p* < 0.05) in two studies [40, 41]. There was a significant difference between depression levels after psychological intervention (*p* < 0.05) in three studies [6, 28, 34]. There was a significant difference between anxiety, depression, and stress level after psychological intervention (*p* < 0.05) in one study [24].

### Quality assessment of each selected study

Among those 26 reviewed studies, 13 RCTs; 12 quasi-experiment design; and 1- one group pre posttest design were included. Among 26 studies, one studies have strong rating (no weak score, all strong score) [26], 24 studies have all moderate score/ strong score (no weak score) and 1 study has weak rating with having more than two weak score [47]. (Refer to Table 3)

**Table 3:** Global rating of reviewed studies for quality assessment

Study N.	Selection bias	Study design	Confounders	Blinding	Data collection methods	Dropouts	Intervention integrity	Analysis	Global rating of paper
1	2	2	2	2	1	1	1	1	Strong
2	2	2	2	2	1	1	1	1	Strong
3	1	1	1	2	1	1	1	1	Strong
4	1	1	1	2	1	1	1	1	Strong
5	1	1	1	2	1	1	1	1	Strong
6	1	1	1	2	1	1	1	1	Strong
7	1	1	1	2	1	1	1	1	Strong
8	1	1	1	2	1	1	1	1	Strong
9	1	1	1	2	1	1	1	1	Strong
10	1	1	1	2	1	1	1	1	Strong
11	1	1	1	2	1	1	1	1	Strong
12	1	1	1	2	1	1	1	1	Strong
13	1	1	1	2	1	1	1	1	Strong
14	1	1	1	2	1	1	1	1	Strong
15	1	1	1	1	1	1	1	1	Strong
16	1	2	1	1	1	1	1	1	Strong
17	2	2	2	2	1	1	1	1	Strong
18	2	2	2	2	1	1	1	1	Strong
19	2	2	2	2	1	1	1	1	Strong
20	2	3	3	3	1	1	1	1	Weak
21	2	2	2	2	1	1	1	1	Strong
22	2	2	2	2	1	1	1	1	Strong
23	2	2	2	2	1	1	1	1	Strong
24	2	2	2	2	1	1	1	1	Strong
25	2	2	2	2	1	1	1	1	Strong
26	2	2	2	2	1	1	1	1	Strong

*GLOBAL RATING FOR PAPER = 1; STRONG (no WEAK ratings), 2; MODERATE (one WEAK rating) and 3; WEAK ((two or more WEAK ratings)*

#### Meta-analysis

**Fig. 2:**
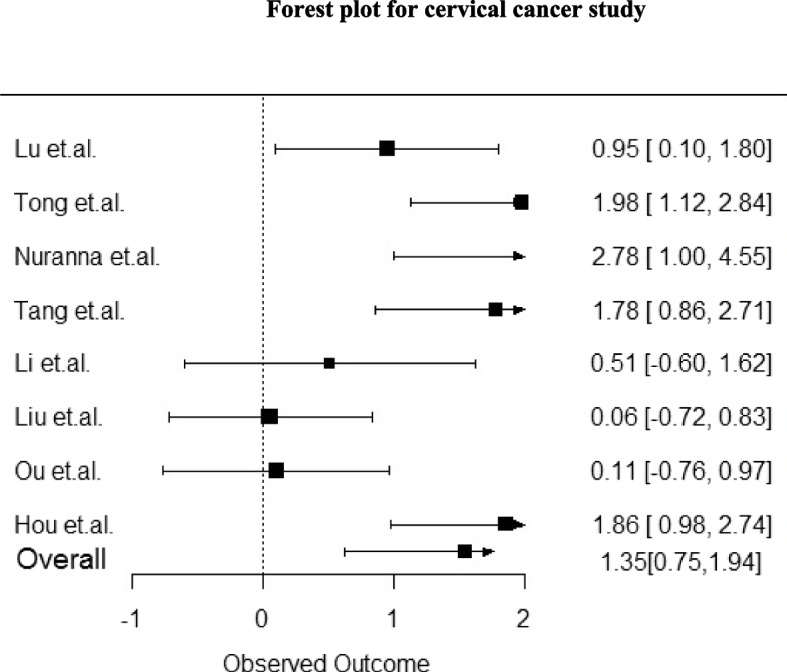
Forest plot of psychological supportive care intervention versus conventional control in alleviating anxiety and depression symptoms

Among twenty-six reviewed studies, eight studies [23, 25–28, 37, 40, 46] were used in meta-analysis on the basis of most homogeneous properties. The meta-analysis evaluated the effect sizes of psychological supportive care interventions. The overall effect size was moderate-to-high (1.35), with a 95% confidence interval of 0.75 to 1.94. It suggests a statistically significant positive effect of the interventions under investigation across the included studies. However, there was some variation in the effect sizes across the studies. Three of the studies (Tong et al., 2021; Nuranna et al., 2018; and Hou et al., 2021) exhibited high effect sizes, suggesting a substantial impact of the studied factors. The other two studies (Lu et al. 2022 and Tang et al. 2022) had medium effect sizes. The low effect size observed in Liu et al. (2021) study indicates a less substantial impact—the wide confidence interval in the study by Nuranna et al. (2018) suggests substantial variability and uncertainty in the estimated effect size, which should be interpreted cautiously. The total sample size of 936 participants is relatively large for meta-analysis, enhancing the reliability of the meta-analysis. Overall, the meta-analysis suggests a moderate-to-high overall effect size for the interventions related to cervical cancer in the included studies, with some studies demonstrating a high impact. However, the wide confidence interval in one study indicates the need for further research and caution in interpreting its results. In Sum, this meta-analysis provides preliminary evidence that the interventions studied in the included research articles may have a positive effect on CC prevention or treatment. However, more research is needed to confirm these findings and to determine the long-term effects of these interventions or outcomes (Refer to Table 4). The forest plot of the meta-analysis is shown in figure and it visually represents individual study effect sizes and the combined effect size (Refer to Fig. 2).

**Table 4:** Study used for meta-analysis

SN	Study	Sample Size (n)	Effect Size	95% CI	Weight (%)
1	Lu et al. (2022)	110	0.951	0.573–1.329	14.09
2	Tong et al. (2021)	86	1.981	1.602–2.36	11.02
3	Nuranna et al. (2018)	32	2.776	0.394–19.568	4.10
4	Tang et al. (2022)	80	1.784	1.338–2.23	14.17
5	Li et.al.(2021)	151	0.511	0.189–0.832	14.09
6	Liu et al. (2021)	141	0.057	-0.279-0.393	14.35
7	Ou et al. (2021)	104	0.107	-0.282-0.496	14.09
8	Hou et.al.(2021)	232	1.859	1.6-2.118	14.09
Total		936	**1.346217893**	0.751–1.942	**100.00**

*Choen’s d test was applied for size effect measurement*

## Discussion

This systematic review aimed to identify the different types of interventions to address the psychological supportive care needs “anxiety and depression” of cervical cancer patients. Overall studies result from various countries had shown the beneficial effect of psychological supportive care intervention and revealed decrease in anxiety and depression level with the use of psychological supportive care intervention among cervical cancer patients.

In addition, the intervention had also a positive effect on quality of life, cortisol level, self-efficacy, sexual function, sleep status, and fatigue.

Our review resulted that the mean age of the respondents was 34.15 ± 10.18 to 66.7 ± 4.5 in the intervention group and between 36.57 ± 11.42 to 65.7 ± 4.1 in the control group by excluding seven studies where the mean age of the respondents did not mention. The mean and SD invite the reader to determine the normal range and think of it as covering most of the distribution of values and the presentation of SD is required in calculations of sample size for approximately normally distributed outcomes and can be used by readers in planning future studies [49].

This review found that, 61.53% of the studies used equal sample sizes in both intervention and control groups, and in other remaining studies, the sample size is larger in the intervention group than in the control group. Equal sample sizes in intervention and control groups maximize the statistical power. The reason for unequal group size is the result of simple randomization, dropouts, and planned differences in group size. Unequal sample size may affect statistical power and type I error rates. The use of larger control groups may give more power to studies looking for an effect in the mid-range but not for large or small effects [50].

This review demonstrated that, heterogeneity was found in different aspects of results: design, measurement instrument type of intervention, intervention delivery technique, duration of intervention, sessions of interventions, follow up period etc. Heterogeneity can make the sense to which the extent that the reviewed studies grasp into same population effect size. The conclusion of zero heterogeneity will come in case of observed differences do not go beyond the expected outcomes due to sampling error [51].

Our review result showed that different health education methods (such as exercise, telephone counselling, educational brochure, family education, consultation sessions, lecture presentations, Self -learning package, face-to-face interviews, medication, psychotherapy, nursing support) are effective in addressing psychological supportive care needs “anxiety and depression” of cervical cancer patients. These findings are consistent with the findings of a systematic review entitled educational interventions for cervical cancer screening [52]. As per the findings of our review traditional chines medicine was used by two for the management of psychological supportive care needs “anxiety and depression” of cervical cancer patients. The efficacy of herbal medicine was found good in a systematic and meta-analysis study among cervical cancer patients for the reduction of cervical cancer toxicity [53].

This review resulted that, the providers of intervention were nursing staff in ten studies. A review study found that nurse-led interventions improve specific cancer-related symptoms, including psychological morbidity [54].

This review showed that mindfulness-based intervention can be used for the reduction of psychological supportive care needs, “anxiety and depression”, and anxiety and depression was significantly reduced in the intervention group. Findings of a systematic review and meta-analysis study suggest that mindfulness-based stress reduction had a significant effect on depressive symptoms (*P* < 0.001) [55].

This review found that physical exercise interventions are useful for the reduction of psychological supportive care needs, “anxiety and depression” and anxiety and depression were significantly reduced in the intervention group. One systematic review on effects of physical exercise interventions for individuals with gynecologic cancer found that cervical cancer is one of the most common gynecological cancer and physical exercise interventions may have beneficial effects on depression and anxiety of this patient population [56]. The findings of another systematic review on home-based aerobic and resistance exercise interventions study identified better outcomes for the reduction of anxiety and depression but there was no significant difference compared with usual care [57]. Mind-body exercise resulted a statistically significant effect on the outcomes of depression, anxiety, (*p* < 0.05) among cancer survivors [58]. Exercise has modest positive effects on depressive symptoms among cancer survivors with larger effects for programs that were supervised or partially supervised, not performed at home [59].

This review found that psychotherapy drug (Racemic Ketamine) without co-intervention resulted in significantly lower HAMD-17 scores than all other groups at 1 day and 3 days postoperatively among cervical cancer patients. Two meta-analysis further support the notion that psychotherapy drug (Racemic Ketamine/ ketamin) without co-intervention resulted in reduction of depression and anxiety: The effect sizes for depression severity, response and remission rates, were statistically greater for racemic ketamine than ketamine. The more effectiveness was found in higher doses than lower doses. Variances were apparent in initial effects, ongoing treatment, and lasting effects after the final dose [60]. Ketamine seems to offer fast and sustained relief from anxiety symptoms across a range of clinical settings, with anxiolytic effects occurring within the first 12 h of administration and remaining effective for one to two weeks [61].

In this review, the majority (ten) of the studies used SDS and SAS for the measurement of anxiety and depression. A result of a review on mindfulness-based stress reduction found that HADS was used as a measurement scale for anxiety and depression by the majority (eight) of the studies [55].

Most of the studies of this review did not mention about homework session. According to Kazantzis and L’Abate 2007; Lambert et al. 2007 homework are activities carried out outside of therapy for the increments of skills which helps to generalize these particular activities with the natural environment [62]. Two meta-analyses explained that greater homework engagement is associated with better treatment outcomes in depression and anxiety [63, 64].

Among twenty-six studies of this review, more than half studies just mentioned the name of the intervention and did not mention in details about the total sessions, duration and frequency of intervention. Explanation regarding details of intervention including total sessions, duration and frequency of intervention give more clear pictures to the reader, ensure that all participants had the same number of sessions and that each session was the same length and increase the quality of paper by removing methodological biasness [65].

None of the studies of this review mentioned about the intervention fidelity measurement. Fidelity is a process necessary for evaluating the efficacy of intervention approaches. Fidelity measurement watch over against deviations from, or drift in, the delivery of a targeted intervention. It also differentiates intervention approaches from each other. It ensures the accurate presentation and examination of intervention approach and prevents possibly false conclusions [66].

Studies exhibited variability in effect sizes, with some showing high (Tong et al., 2021; Nuranna et al., 2018; Hou et al., 2021) [23, 26, 28] and medium (Lu et al., 2022; Tang et al., 2022) effects [25, 37]. Liu et al. (2021) reported a low effect size [42]. A wide confidence interval in Nuranna et al.‘s (2018) study urged cautious interpretation [28].

### Strengths and limitation

This is the first systematic review employed to identify the psychological intervention carried out to address psychological supportive care needs, especially anxiety, and depression. This study was conducted according to the Preferred Reporting Items for Systematic Reviews and Meta-Analyses guidelines (PRISMA) and the recommendations of the Cochrane Collaboration. A comprehensive and rigorous literature search was performed. RCTs, quasi-experimental, and one-group pre-posttest designs were included in the study. Selection of studies, data extraction, and risk of bias assessment was done by independent researchers. Standard tools were used for reporting review. The quality of interventional studies was evaluated EPHPP tool. The review includes studies conducted in Asia, America, Europe, and Africa with a large number of 11,638 CC patients which expands the generalizability of findings.

This study did not include the ‘grey’ literature so future studies need to survey this literature. Due to lack of access, only full-text available studies were included in this review, the studies without full text were excluded which might have caused to miss to add essential evidence in this review. The studies available in other language than English were excluded due to difficulties related to translation and funding which also might have missed the collection of relevant studies related to this review.

Due to inclusion limitation, it brings heterogeneity effect and the main areas of heterogeneity are the design, sample size, type of intervention, period of intervention, intervention provider; control group; stage of cervical cancer; and instrument of outcome. So, the general explanation of the findings should be made cautiously. The secondary outcomes such as quality of life, cortisol level, self-efficacy, sexual function, sleep status, and fatigue, were not focused in current review.

### Recommendations for future research

Future studies on systematic reviews need to involve RCTs for quantifying the effectiveness of different psychological supportive care needs interventions. Moreover, future studies need to identify the effect of psychological intervention on other parameters like fatigue, pain, sleep status, and self-efficacy.

### Implications for practice

Despite limitations, the results of these studies have substantial implications for addressing anxiety and depression among CC patients. Anxiety and depression are still big psychological supportive care needs among them in many countries. The integration of psychological supportive care intervention approach in their countries and effective implementation of intervention as reported from this review will help to psychological busting by reducing anxiety and depression among CC patients.

## Conclusions

Psychological supportive care interventions are found in many forms like exercise, counselling, special nursing care, peer education, family education, psychotherapy, medication, etc. All types of psychological intervention are found effective for the reduction of psychological supportive care needs “anxiety and depression” among CC patients. Nurse led psychological supportive care intervention resulted in a high effectiveness of intervention. Homework session will aid in continuity of intervention.

## Data Availability

Corresponding author stores the data fully confidential and safely. We always agree to review the primary data upon the request of the journal.
